# Challenges and barriers in managing Type 2 diabetes: insights from a qualitative study of patients’ experiences at a university hospital in Qassim, Saudi Arabia

**DOI:** 10.1080/16549716.2025.2591543

**Published:** 2026-01-08

**Authors:** Andrew Dumale Ngo, Mohammed Bien Kulintang, Talal Ali F. Alharbi, Rasha Mohammed Hussein, Hani Al-Najjar, Fadhel Majed Alharbi, Metab Algeffari, Yara H. Sawan

**Affiliations:** a Department of Community, Psychiatric, and Mental Health Nursing, College of Nursing, Qassim University, Buraydah, Saudi Arabia; b Department of Nursing Administration and Education, College of Nursing, Shaqra University, Dawadmi, Riyadh, Saudi Arabia; c Nursing Department, Qassim University Medical City, Buraidah, Saudi Arabia; d Department of Family and Community Medicine, College of Medicine, Qassim University, Buraydah, Saudi Arabia; e Abdullah Al-Othaim Diabetes Center, Medical City, Qassim University, Buraydah, Saudi Arabia

**Keywords:** healthcare access, medication adherence, patient education, lifestyle management, cultural influence

## Abstract

**Background:**

Type 2 diabetes is a global health issue, with unique challenges in Qassim, Saudi Arabia. Patients face barriers such as lifestyle, diet, healthcare access, and psychological impacts. Understanding these challenges is crucial for improving diabetes care and support.

**Objective:**

The aim of this study was to explore the challenges and barriers faced by patients in managing Type 2 diabetes at a University Hospital in Qassim, Saudi Arabia, to provide insights for improving diabetes care and support.

**Methods:**

A qualitative descriptive phenomenological design used semi-structured interviews and purposive sampling. Face-to-face interviews (Sept 2024–Jan 2025) were audio recorded and transcribed verbatim. Data were analyzed using Collaizi’s thematic analysis.

**Results:**

This study provides a comprehensive understanding of the key challenges and facilitators influencing the management of Type 2 diabetes. Thematic analysis identified five core themes: *Challenges in Adapting Lifestyle and Behavior, Dietary Struggles and Social Pressures, Barriers to Effective Healthcare and Medication Use, Psychological and Emotional Burden of Diabetes, and Gaps in Patient Education and Support for Self-Management*. Participants faced challenges of balancing self-care with work and social duties, dietary struggles tied to culture, healthcare barriers, emotional distress, and the vital role of education in disease management.

**Conclusion:**

A patient-centered approach is key to managing Type 2 diabetes in Qassim, Saudi Arabia. Addressing lifestyle, healthcare, and psychological challenges can improve self-management and outcomes. Providers should offer tailored education, enhance care access, and support emotional and social needs. Policies should promote culturally sensitive, community-based interventions to improve adherence and quality of life.

## Paper context

- **Main findings**: Patients with Type 2 Diabetes Mellitus (T2DM) encounter major barriers to effective self-management, including occupational stress, cultural dietary practices, limited healthcare access, medication challenges, and emotional burden.
- **Added knowledge**: Social and cultural contexts, including gatherings and religious practices, shape dietary choices and adherence, while individual factors like self-efficacy also influencing outcomes.
- **Global health impact for policy and action**: Holistic, patient-centered interventions that integrate education, healthcare policy improvements, and psychological support are crucial for improving health outcomes.

## Background

Type 2 diabetes mellitus (T2DM) represents a significant public health challenge in Saudi Arabia, a country with one of the highest global prevalence rates of the disease. Over 7 million individuals are affected, and this number continues to grow due to factors such as urbanization, sedentary lifestyles, and cultural dietary patterns [1,2]. The high prevalence of T2DM places immense pressure on the healthcare system, particularly government hospitals, which are primary care providers for the majority of the population. Poor glycemic control among patients is widespread and contributes to severe microvascular and macrovascular complications, further exacerbating the burden on healthcare resources and diminishing quality of life [3].

Patients encounter a complex interplay of challenges that hinder effective diabetes management. These include logistical barriers such as transportation difficulties and limited access to timely appointments, particularly in rural areas [4]. Cultural pressures, sedentary lifestyles, and non-medical factors such as fear of medication side effects (20.4%) and the burden of taking multiple drugs (19.1%) also significantly influence glycemic control and HbA1c improvement [2,5]. Moreover, gaps in patient education, particularly about medication use and potential side effects, remain critical contributors to poor self-management [6]. To address these issues, interventions such as telemedicine and educational initiatives have been introduced, yet their impact has been uneven, highlighting the need for more tailored approaches [2].

Moreover, patients with T2DM often adopt self-initiated strategies to navigate these challenges, including seeking support from healthcare providers, community networks, and online resources. They also rely on family and friends for practical and emotional support, as well as for guidance on medication safety and management. These strategies emphasize the importance of engaging community and social networks in patient-centered interventions to enhance self-management activities [7]. However, significant gaps persist in understanding how these barriers and strategies manifest within the unique sociocultural and healthcare context of Saudi Arabia.

Even though interventions such as telemedicine, diabetes education programs, and lifestyle modification campaigns have been introduced in Saudi Arabia, their impact has been uneven. Urban populations often benefit more due to better access to resources and technology, while rural areas face persistent gaps in care delivery and follow-up [8,9]. Furthermore, these interventions frequently adopt a one-size-fits-all approach that overlooks cultural norms, dietary practices, and social pressures unique to Saudi communities [10,11]. As a result, many patients continue to experience poor glycemic control and low adherence to treatment, underscoring the need for research that explores patients’ lived experiences to inform the development of tailored, culturally sensitive interventions [4,12].

Although existing studies highlight challenges in T2DM, most focus on quantitative outcomes or generalized assessments of care. Few have qualitatively explored patients’ lived experiences in government hospitals, particularly in regions like Qassim, where healthcare access, resources, and cultural norms differ from urban centers. In Qassim, frequent family gatherings, traditional dietary practices emphasizing dates and sweetened foods, and limited public exercise spaces due to climate further intensify barriers to diabetes management. This study addresses these gaps by examining the barriers faced by T2DM patients at a university hospital in Qassim, Saudi Arabia. Using a qualitative approach, it provides nuanced insights into patients’ experiences, self-management, and the influence of healthcare and community support, informing targeted interventions to improve care and outcomes.

### Theoretical framework

**Figure 1.**
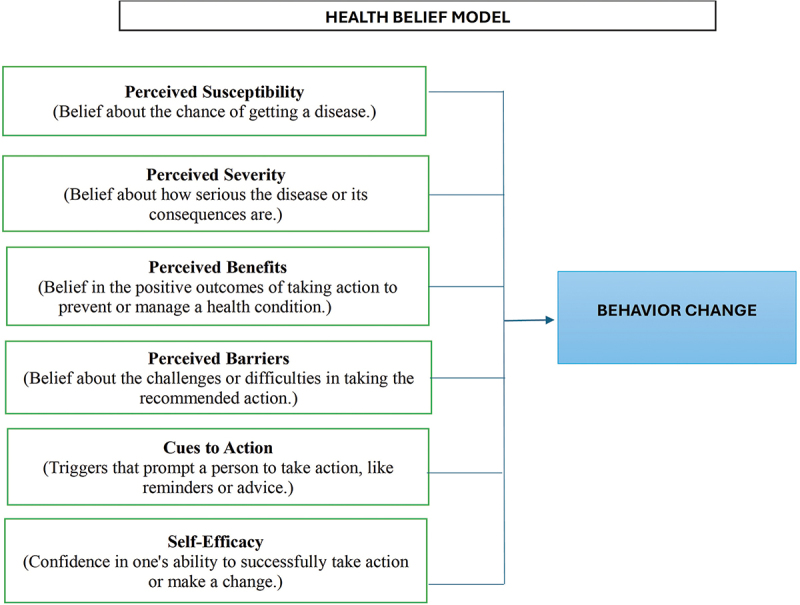
Health Belief Model framework [13].

This study is guided by the Health Belief Model (HBM), which explains how individuals’ health behaviors are shaped by their perceptions of illness. Patients are more likely to engage in positive behaviors when they perceive susceptibility and severity, recognize benefits, and overcome barriers [13]. The model also highlights cues to action and self-efficacy as key drivers of behavior change. In Type 2 diabetes management, it helps examine how these beliefs influence adherence to lifestyle changes, medication, and self-care practices. This relationship is illustrated in Figure 1, showing how the model’s components collectively influence an individual’s motivation and behavior change.

## Methods

This study employed a descriptive phenomenological design guided by the Health Belief Model (HBM) to explore patients’ lived experiences in managing Type 2 diabetes. The HBM focused on perceived risks, barriers, and self-efficacy [13], while Colaizzi’s method guided data analysis [14]. The study aimed to examine challenges faced by patients at a university hospital in Qassim, Saudi Arabia, considering personal, systemic, and sociocultural factors. COREQ guidelines [15] were followed to ensure methodological rigor.

Participants were purposively selected for diverse representation. Inclusion criteria were adults (≥18 years) with Type 2 diabetes for ≥1 year, receiving treatment, able to communicate in Arabic, and willing to consent. Exclusion criteria included Type 1 or gestational diabetes, diagnosis <1 year, not receiving hospital treatment, or cognitive impairments.

### Data collection

Data collection was conducted between September 2024 and January 2025 at a university hospital in Qassim, Saudi Arabia, which houses one of the region’s main specialized diabetes centers. The site was selected despite the presence of other hospitals because it provides comprehensive care and education to a large and diverse population, serving up to 1,267 patients. This high patient volume made the hospital an appropriate and representative setting for exploring challenges in Type 2 diabetes management.

Participants were recruited purposively from the outpatient diabetes clinic, with eligible patients identified through clinic schedules in collaboration with staff. Recruitment and interviews were conducted by two researchers after explaining the study purpose and eligibility criteria. Written informed consent was obtained. In total, 20 patients participated, while two declined due to time constraints.

All interviews were conducted in Arabic by a physician and nurse experienced in diabetes care and qualitative research. As some participants were also their patients, the interviewers acknowledged this prior clinical relationship as a potential bias. To minimize its influence, they applied bracketing, maintained neutrality, reassured participants their responses would not affect care, and engaged in peer debriefing – discussing with a colleague how prior knowledge might influence questioning and adjusting their approach to ensure fair, consistent data collection. Interviews took place in private hospital rooms, lasted 30–60 min, were audio-recorded with permission, and supplemented with field notes on non-verbal cues (e.g. pauses, tone, lack of eye contact) and contextual observations such as stress when discussing medication side effects or frustration with dietary restrictions.

The semi-structured interview guide was developed after reviewing relevant literature on diabetes and qualitative research. To ensure clarity and cultural relevance, it was reviewed by two experts in diabetes care and one qualitative researcher. A pilot with two participants showed that some questions were hard to understand and answers were brief, highlighting the need to simplify wording and incorporate probing questions to encourage more detailed and meaningful narratives. The final guide was prepared in both English and Arabic and validated by the expert reviewers. It comprised four sections with 12 core questions: (1) background and daily life (three questions); (2) healthcare experiences (three questions); (3) treatment and self-management (four questions); and (4) psychological and social aspects (two questions). Sample open-ended questions included What challenges do you face in managing diabetes?; How do daily responsibilities affect self-care?; What difficulties arise in following dietary recommendations?; How has diabetes affected you emotionally? and What changes would you suggest to better support patients with Type 2 diabetes?

Recordings were transcribed verbatim in Arabic and translated into English by bilingual team members with medical translation experience. Accuracy was ensured through back-translation by an independent professional, with discrepancies resolved by consensus. Rigor was maintained through member checking, prolonged engagement, triangulation with field notes, and rich contextual descriptions to enhance transferability. Dependability was supported by an audit trail and systematic coding with Atlas.ti, while confirmability was reinforced through bracketing, peer debriefing, and independent coding. Data saturation was reached after 18 interviews, with two additional interviews confirming no new themes. No financial incentives were provided, though participants received educational materials and were assured findings would help improve hospital services.

### Data analysis

Data were transcribed from Arabic into English and analysed using Colaizzi’s thematic approach [14]. Significant statements were extracted, meanings formulated, and themes and subthemes developed to capture participants’ experiences. Findings were validated with participants to ensure accuracy. Two researchers independently coded the data, resolving discrepancies by consensus and organized responses using a coding tree in Atlas.ti. Trustworthiness was enhanced through participant validation and the use of direct quotations to illustrate key themes.

## Results

**Figure 2.**
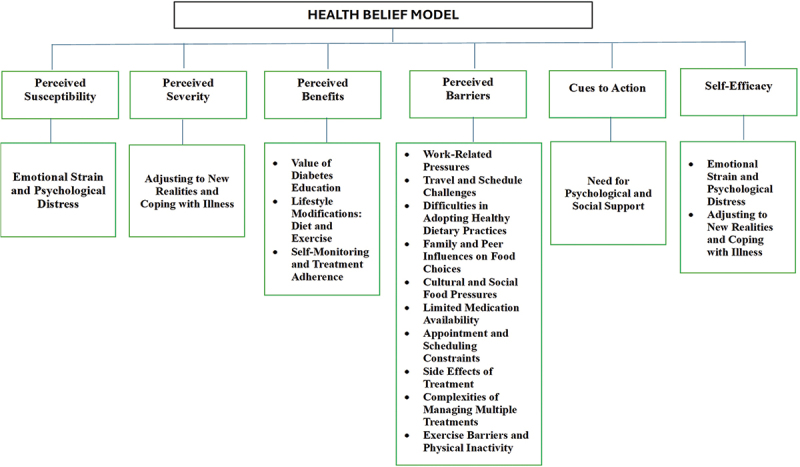
Alignment of study subthemes with Health Belief Model constructs.

Thematic analysis of 280 statements yielded five core themes and 16 subthemes highlighting key challenges in managing Type 2 diabetes. These themes capture the most significant aspects of participants’ experiences and factors influencing management. Table 1 shows participant demographics, Table 2 presents themes and subthemes, and Figure 2 maps subthemes onto Health Belief Model constructs. Focusing on subthemes provides greater specificity and links lived experiences to the theoretical framework, offering nuanced insights for action.

**Table 1.** Demographic characteristics of the participants (*n* = 20).

Age	Sex	Occupation	Educational Level	Marital Status	Years with Diabetes
53	M	Asst. Professor	PHD	Married	15
48	M	Asst. Professor	PhD	Married	14
58	M	Employee (Ministry of Justice)	Bachelors	Married	3
41	M	Self-Employed	High School Graduate	Separated	15
47	M	Driver	High School	Married	6
48	F	Administrator	Bachelors	Married	8
52	M	Nutrition Technician	Diploma in Nutrition	Married	15
48	M	Bank Employee	Diploma	Married	7
42	M	University Employee	High School	Single	16
58	M	Asst. Prof	PHD	Married	23
58	M	Self-Employed	Bachelor’s	Married	8
61	M	Retired	High School	Married	16
52	M	Engineer	Bachelors	Married	4
49	M	University Security	Middle School	Married	12
48	M	Assistant Professor	PHD	Married	10
53	M	Project Manager	High School	Married	15
30	M	Student	Masters	Married	4
68	M	Unemployed	High School	Married	15
40	M	Administrator	High School	Married	4
66	M	Teacher	Bachelors	Married	25

**Table 2.** Core themes and subthemes.

Core Theme	Subthemes
1. Challenges in Adapting Lifestyle and Behavior	1.1 Work-Related Pressures
	1.2 Travel and Schedule Challenges
	1.3 Exercise Barriers and Physical Inactivity
2. Dietary Struggles and Social Pressures	2.1 Difficulties in Adopting Healthy Dietary Practices
	2.2 Family and Peer Influences on Food Choices
	2.3 Cultural and Social Food Pressures
3. Barriers to Effective Healthcare and Medication Use	3.1 Limited Medication Availability
	3.2 Appointment and Scheduling Constraints
	3.3 Side Effects of Treatment
	3.4 Complexities of Managing Multiple Treatments
4. Psychological and Emotional Burden of Diabetes	4.1 Emotional Strain and Psychological Distress
	4.2 Adjusting to New Realities and Coping with Illness
5. Gaps in Patient Education and Support for Self-Management	5.1 Value of Diabetes Education
	5.2 Lifestyle Modifications: Diet and Exercise
	5.3 Self-Monitoring and Treatment Adherence
	5.4 Need for Psychological and Social Support

### Challenges in adapting lifestyle and behavior

Managing diabetes requires major lifestyle adjustments, often complicated by *work-related pressures, travel and schedule challenges, and exercise barriers and physical inactivity*. Participants reported stress, missed doses, and difficulties maintaining treatment. One shared, ‘Busy routine with exams and research leaves no time for exercise and causes stress-induced high blood sugar’ (Participant 18, SS61), while another noted, ‘Long work hours forced me to take medications late, causing energy drops at night’ (Participant 10, SS13 & SS53).

Travel and shifting schedules also disrupted management. One participant explained, ‘When I travel, my treatment is interrupted because I can only buy medication from the country, I’m in’ (Participant 16, SS86). Another added, ‘My routine is not fixed due to my freelance work … This lack of a clear routine could be the main reason I sometimes struggle with my treatments’ (Participant 11, SS79).

Exercise barriers were common, often due to time constraints and lack of suitable spaces. One noted, ‘Reduced physical activity after moving to Saudi Arabia compared to Sudan affected me’ (Participant 18, SS21), while another said, ‘My doctor advises me to walk for half an hour, but due to my schedule, physical activity is very difficult, and I don’t have time or energy for it’ (Participant 17, SS194).

### Dietary struggles and social pressures

Patients with diabetes often struggle to balance social life and dietary requirements due to *challenges in adopting healthy practices, family and peer influences, and cultural and social food pressures*. Many reported difficulties following dietary guidelines because of cravings, fatigue, or limited healthy options. One participant shared, ‘I feel the urge to eat indulgent foods and crave rich, fatty meals. Psychological pressure and fatigue were challenges when starting a healthy lifestyle’ (Participant 13, SS56). Another noted, ‘Difficulty avoiding sweets at gatherings and eating restaurant food due to being single’ (Participant 1, SS199).

Family and peer influences also undermined self-discipline, as friends and relatives encouraged indulgence. One participant explained, ‘Socializing daily with friends makes managing diabetes hard due to sweets and dates served with coffee’ (Participant 20, SS64). Another said, ‘Food advertisements for fast food reach my children, and I feel compelled to take them to restaurants. Gatherings with family and friends involve rich and delicious food, with invitations I can’t decline’ (Participant 2, SS200–201).

Cultural and social food pressures further complicated management, as hospitality traditions often involve high-sugar meals. One participant stated, ‘It is difficult to refuse personal invitations, especially since they often involve a lot of food leading to increased blood sugar’ (Participant 1, SS199). Another added, ‘In Arab cultures, our hospitality often involves sugary foods such as dates, commonly offered as a gesture of welcome’ (Participant 10, SS210).

### Barriers to effective healthcare and medication use

Patients face challenges in diabetes management, including *limited medication availability, appointment and scheduling constraints, side effects of treatment, and complexities of managing multiple treatments*. Some struggled to find medications due to stock shortages: ‘Sometimes, I have to search for days to find certain medications at pharmacies’ (Participant 19, SS89). Appointment timing was also difficult: ‘The timing of appointments is particularly draining, especially when it’s after work hours’ (Participant 14, SS139). Treatment side effects affected adherence, as one participant explained, ‘The weekly injections caused me constipation, so I started taking them every 10 days instead of weekly’ (Participant 12, SS80). Managing multiple medications further complicated adherence: ‘The number of medications and forgetfulness make it difficult to adhere to the treatment plan’ (Participant 7, SS74).

### Psychological and emotional burden of diabetes

The psychological burden of diabetes management was evident, with participants reporting *emotional strain and psychological distress and adjusting to new realities and coping with illness*. Many expressed stress and anxiety over constant monitoring and medication routines, with one stating, ‘Stress, irritability, and mood swings affect me to the point that I reach a state of severe anxiety’ (Participant 6, SS127). Over time, some adapted to their condition, developing coping mechanisms and resilience. One participant reflected, ‘I went through a period of depression that kept me at home. Now I’m fine, and nothing affects my well-being’ (Participant 3, SS124).

### Gaps in patient education and support for self-management

Participants emphasized the *value of diabetes education, lifestyle modifications: diet and exercise, self-monitoring and treatment adherence, and need for psychological and social support* in managing their condition. Education was seen as essential, with one participant stating, ‘Activating diabetes education is crucial and has positively reflected on my health’ (Participant 4, SS261). Many stressed the importance of a balanced diet and exercise, noting that medication alone is insufficient: ‘For diabetic patients, I recommend treatment along with proper eating; treatment without food is never effective’ (Participant 3, SS259).

Self-monitoring and routine adherence were highlighted, with one participant noting, ‘Enhancing self-monitoring significantly improves Type 2 diabetes management’ (Participant 17, SS276). Psychological and social support also helped participants cope, as one shared, ‘Avoiding stress and not isolating myself at home because of diabetes are important’ (Participant 5, SS262), while another added, ‘The patient must understand what changes have taken place and learn how to manage the issue and reduce the likelihood of its progression’ (Participant 10, SS269).

## Discussion

Managing diabetes requires major lifestyle changes, often complicated by work demands, irregular schedules, and limited opportunities for physical activity. Participants described stress from professional responsibilities that disrupted self-care routines, echoing findings that work-related stress impairs effective glycemic control [16]. Long hours and shifting schedules also limited physical activity, a challenge reinforced in prior research linking work conditions, workload, and poor job control with weaker diabetes self-management [17]. Addressing these occupational and environmental barriers is essential to help patients maintain consistent routines and physical activity. Although our participants described clear links between occupational demands, stress, and disrupted routines, evidence from broader populations is mixed. Some studies found no direct association between psychosocial work stress and glycemic control, suggesting that stress alone may not universally impair management. Similarly, long working hours did not consistently increase diabetes risk, with effects varying by socioeconomic status and job type. These findings suggest that while work strain may hinder management, occupational influences are complex, warranting further study of factors like shift patterns, sleep, and job control [18,19].

Food-related challenges were shaped by cultural and social contexts. Participants struggled to resist high-sugar and high-fat foods, especially during social gatherings. Similar findings in Moroccan and African American communities show how cultural norms and peer pressure can undermine adherence [10,20]. These results highlight the need for culturally tailored education and community strategies, with social network interventions showing promise in reshaping group behaviors [21]. In contrast, some studies suggest individual factors may be stronger drivers of lapses. Ecological momentary assessments found that cravings, fatigue, and eating outside the home often triggered lapses more than social pressure [22]. Self-efficacy also predicts better adherence and glycemic outcomes, outweighing external influences [23]. Thus, while cultural pressures matter, interventions must also strengthen self-regulation and coping skills.

Healthcare access emerged as a critical barrier, with stock shortages, long appointment intervals, and inconvenient schedules disrupting care. These challenges align with evidence showing that limited healthcare access contributes to diabetes complications [24]. Medication complexity, side effects, and polypharmacy further undermined adherence, with global nonadherence rates estimated at 30% to 50% [25]. Similar problems in affordability and accessibility have been documented in Ethiopia and Iran, underscoring the need for stronger supply chains, wider insurance coverage, and stricter cost regulation [9,26]). Simplified treatment regimens and better follow-up have been suggested as strategies to enhance adherence and outcomes. In contrast, access alone does not fully explain poor adherence; patients’ beliefs, health literacy, and cultural factors often play a stronger role [27,28]. Even when medicines are available, knowledge, social support, and attitudes influence adherence [29]. Integrating WhatsApp-based diabetes self-management education can improve self-efficacy and health outcomes in people with T2DM and should be prioritized in clinics [30]. Together, these findings suggest that alongside improving access, strengthening patient knowledge, beliefs, and digital education is key to sustaining long-term adherence.

Participants frequently described emotional strain, including stress, anxiety, and episodes of depression, all of which interfered with self-monitoring and adherence. This aligns with research showing that psychological distress is a major predictor of non-adherence in type 2 diabetes [31]. Conversely, self-efficacy, social support, positive affect, and effective emotion regulation improve coping, disease management, and long-term blood glucose control [32–34]. These findings underscore the need to integrate psychological support into diabetes care, with psychologists and counselors playing an active role in strengthening resilience, promoting self-efficacy, and targeting emotional well-being to optimize both glycemic and psychosocial outcomes [35].

Despite these barriers, participants highlighted the benefits of diabetes education in strengthening self-care, improving BMI, glycemic control, medication adherence, and self-management knowledge [12,36]. Calls for more accessible, ongoing education reinforce the need for holistic, patient-centered approaches combining medical, lifestyle, social, and psychological support [8]. Supporting this, adults receiving 2 years of frequent structured education followed by annual reinforcement maintained good glycemic control, with frequent SMBG linked to better long-term outcomes [37]. Structured, culturally tailored DSME programs have also been shown to improve self-care, glycemic control, and cardiovascular markers [38,39]. In contrast, low-intensity interventions targeting medication adherence alone were largely ineffective [40], highlighting that intensity, structure, and patient-centered strategies are critical for meaningful outcomes.

While evidence on lifestyle, social, and healthcare barriers in diabetes management is mixed, our findings show these challenges are particularly relevant in our hospital context. Cultural and social factors in Qassim–such as dietary norms favoring large portions and sweetened foods, frequent family gatherings, and hot weather limiting outdoor activity–help explain why patients often struggle to sustain lifestyle changes despite awareness and willingness. These realities underscore the need for hospital-level strategies, including flexible clinic hours, reliable medication supply, psychosocial support, and culturally tailored education, to complement individual efforts. While challenges in T2DM management are shared globally, our results emphasize that interventions must be localized to address the specific sociocultural realities of patients in Qassim.

### Strengths and limitations

This study explores the challenges of managing Type 2 diabetes in Qassim, Saudi Arabia, offering context-specific insights. Colaizzi’s thematic analysis provided a deep understanding of personal, social, and healthcare barriers, with purposive sampling ensuring diverse perspectives. Some themes were validated by participants. However, findings are limited by a single hospital setting, potential recall and selection biases, and interviewer influence. Further research in varied settings is needed to expand these findings.

## Conclusions

Managing Type 2 diabetes in Qassim is complex, shaped by lifestyle, social influences, healthcare access, psychological well-being, and education. A patient-centered approach that integrates education, healthcare improvements, and psychosocial support is essential to overcoming the barriers to diabetes management. Addressing these challenges through tailored interventions can enhance self-efficacy, improve adherence, and promote better health outcomes. Future research should focus on exploring culturally relevant interventions, while healthcare providers must adopt flexible and personalized strategies to improve the quality of life for patients and ensure sustained adherence to treatment.

## Supplementary Material

COREQ_Checklist.pdf

## Data Availability

Dataset is available from the corresponding author upon reasonable request, due to the sensitive and confidential nature of the qualitative data.
